# Leveraging Large-Scale Public Data for Artificial Intelligence-Driven Chest X-Ray Analysis and Diagnosis

**DOI:** 10.3390/diagnostics16010146

**Published:** 2026-01-01

**Authors:** Farzeen Khalid Khan, Waleed Bin Tahir, Mu Sook Lee, Jin Young Kim, Shi Sub Byon, Sun-Woo Pi, Byoung-Dai Lee

**Affiliations:** 1 AI Laboratory, HealthHub Co., Ltd., Seoul 06524, Republic of Korea; 2 Department of Radiology, Keimyung University Dongsan Hospital, Daegu 24601, Republic of Korea; 3 Division of AI and Computer Engineering, Kyonggi University, Suwon 16227, Republic of Korea

**Keywords:** artificial intelligence, chest X-ray interpretation, deep learning, noisy labels, uncertainty quantification

## Abstract

**Background**: Chest X-ray (CXR) imaging is crucial for diagnosing thoracic abnormalities; however, the rising demand burdens radiologists, particularly in resource-limited settings. **Method**: We used large-scale, diverse public CXR datasets with noisy labels to train general-purpose deep learning models (ResNet, DenseNet, EfficientNet, and DLAD-10) for multi-label classification of thoracic conditions. Uncertainty quantification was incorporated to assess model reliability. Performance was evaluated on both internal and external validation sets, with analyses of data scale, diversity, and fine-tuning effects. **Result**: EfficientNet achieved the highest overall area under the receiver operating characteristic curve (0.8944) with improved sensitivity and F1-score. Moreover, as training data volume increased—particularly using multi-source datasets—both diagnostic performance and generalizability were enhanced. Although larger datasets reduced predictive uncertainty, conditions such as tuberculosis remained challenging due to limited high-quality samples. **Conclusions**: General-purpose deep learning models can achieve robust CXR diagnostic performance when trained on large-scale, diverse public datasets despite noisy labels. However, further targeted strategies are needed for underrepresented conditions.

## 1. Introduction

Chest X-ray (CXR) is an essential diagnostic tool in healthcare, providing a cost-effective, non-invasive, and widely accessible method for detecting thoracic diseases [1,2,3]. However, the increasing demand for CXR studies has placed a burden on radiologists, leading to diagnostic delays and highlighting the need for scalable automated diagnostic solutions to enhance efficiency and accuracy [4]. Although deep learning has achieved radiologist-level performance in CXR interpretation [5,6,7,8], its clinical integration is hampered by the reliance on private, expert-annotated datasets that limit reproducibility—especially in resource-constrained settings. Public datasets, including National Institute of Health (NIH) Chest X-ray-14 [9], CheXpert [10], and Medical Information Mart for Intensive Care-CXR (MIMIC-CXR) [11], offer diverse training data; however, noisy labels pose challenges for model reliability [12].

In this study, we aimed to demonstrate that the scale and diversity of training data can offset imperfections in label quality and model specificity. We leveraged large-scale, diverse public CXR datasets to train general-purpose image classification models that robustly classify multiple thoracic conditions without the need for specialized architectures. Moreover, we incorporated uncertainty quantification (UQ) to assess predictive performance and model reliability, offering insights into their real-world clinical applicability. Our findings indicate that, despite noisy labels, general-purpose deep learning models can achieve acceptable diagnostic performance when trained on expansive, varied datasets, thereby supporting the development of accessible, scalable artificial intelligence (AI) diagnostic tools for resource-limited settings.

While our study leverages publicly available datasets and standard deep learning models, its novelty lies in the systematic integration and analysis of these components. Specifically, we:

- Aggregate and harmonize 17 public CXR datasets, addressing label inconsistencies and cross-dataset duplication to enable robust generalization analysis and broaden coverage of rare diseases.
- Perform comprehensive UQ across multiple dataset scales, providing new insights into model stability, calibration, and reliability beyond what prior CXR studies have reported.
- Conduct class-specific uncertainty analysis for clinically important but underexplored categories, including tuberculosis, pneumothorax, masses/nodules, and the heterogeneous “Other” class, offering a granular understanding of class-dependent behavior.
- Characterize scaling-law behavior in both performance and uncertainty, demonstrating how increasing dataset size affects diagnostic accuracy and epistemic uncertainty, and offering actionable guidance for data collection and clinical deployment strategies.

These contributions underscore that our research extends beyond incremental improvements, providing new methodological and practical insights for scalable, uncertainty-aware CXR analysis.

## 2. Materials and Methods

The Institutional Review Board of Keimyung University Dongsan Medical Center approved this retrospective study and waived the requirement for written informed consent. All procedures followed the approved protocol.

### 2.1. Target Conditions

To maximize clinical relevance and impact, this study focused on the following six prevalent and clinically significant thoracic conditions: pneumonia, pleural effusion, tuberculosis, mass, consolidation, and pneumothorax. These conditions include both specific diseases (e.g., pneumonia and tuberculosis) and radiological findings (e.g., pleural effusion, mass, and consolidation) that frequently indicate an underlying pathology. The selection was guided by global disease burden, potential for early intervention, and the diagnostic importance of CXR in routine clinical workflows. Pneumonia and tuberculosis remain the leading causes of morbidity and mortality, particularly in low-resource settings with limited diagnostic tools [13]. Pleural effusion, masses, and consolidation are common thoracic abnormalities that, if detected early, can alter patient management and improve outcomes. Pneumothorax, although less prevalent, is a life-threatening condition requiring immediate identification and intervention [14]. For cases involving abnormal findings not otherwise classified among these six target diseases, an “Other” disease category was included to ensure broad diagnostic coverage without compromising specificity. This strategy enhances the clinical utility while maintaining focus on conditions with the greatest potential impact on patient care.

### 2.2. Data Collection and Preparation

#### 2.2.1. Development Dataset

To construct a comprehensive training dataset, we aggregated 17 public CXR datasets (Table 1) into 894,373 unique frontal-view images, excluding non-frontal projections to ensure clinical consistency. This strategy enhanced model generalizability by incorporating diverse patient demographics, imaging protocols, and disease prevalence. Five major datasets (NIH Chest X-Ray-14 [9], CheXpert [10], MIMIC-CXR [11], PadChest [15], and BRAX [16]) provided a substantial portion of the development dataset. These datasets employed natural language processing (NLP) tools or deep learning algorithms to automatically extract labels from radiological reports. These methods facilitate large-scale label generation and introduce variability in label quality, particularly in disease categories requiring subjective interpretation. To address this, we harmonized labels across datasets, treated low-confidence labels consistently, and ensured mutual exclusivity in training and test sets (see Appendix B for full details).

Table 1 highlights class imbalance—a pervasive challenge in public CXR datasets—across target diseases. To address this, we strategically adjusted per-disease sample counts and augmented underrepresented classes (tuberculosis, mass, consolidation, pneumothorax) via image duplication and online augmentation techniques. Augmentation was restricted to training and validation sets to preserve the integrity of the uniquely composed test set, thereby preventing data leakage. Table 2 provides the final class distribution across all dataset splits.

#### 2.2.2. External Validation Dataset

To assess generalization and fine-tuning efficacy, we constructed an independent validation dataset of 3031 unique-patient, posterior–anterior CXR images (July 2017–December 2019) from our hospital’s picture archiving and communication system (Table 2), ensuring no overlap with training data. This dataset enabled (1) an unbiased assessment of baseline model performance and (2) evaluation of domain-specific fine-tuning. To evaluate domain-specific fine-tuning, we used 10-fold cross-validation (training on k−1 folds and validating on the remaining fold), whereas non-fine-tuned models were directly tested on the external set to isolate domain adaptation effects.

### 2.3. Deep Learning Models

The three deep learning models used in this study—ResNet-50 [28], DenseNet-121 [29], and EfficientNet-B5 [30]—were selected for their proven effectiveness in natural image classification tasks and served as robust baselines for comparison. Furthermore, Deep Learning-based Algorithm for Detecting 10 Abnormalities (DLAD-10) [5], a high-performance model designed for CXR classification and recognized as a base model in commercial services due to its superior performance, was also included in the experiment.

All classification models were initialized with ImageNet-pretrained weights, and their inputs were normalized using the standard ImageNet mean and standard deviation. This preprocessing preserves the activation scaling expected by pretrained backbones and contributes to stable optimization during fine-tuning. In contrast, the U-Net segmentation model used to extract lung masks was trained entirely from scratch, without ImageNet initialization; therefore, segmentation inputs were processed using only min–max intensity normalization, ensuring consistency between preprocessing and initialization strategies.

For classification experiments, all models were trained and evaluated on the same pooled dataset to ensure consistent comparisons. Training was performed using the Adam optimizer with an initial learning rate of 1 × 10^−3^ and a batch size of 32. A comprehensive list of all hyperparameters, ensuring full reproducibility, is provided in Appendix A.

Although more recent architectures—including Vision Transformers [31], ConvNeXt [32], and large foundation models [33]—have demonstrated strong performance in medical image analysis, they require substantially greater computational resources and specialized pretraining pipelines. Consistent with our objective of evaluating scalable and broadly deployable diagnostic models, we focused on representative convolutional architectures that are widely used, computationally accessible, and well supported in open-source frameworks. This design choice is particularly relevant for resource-limited clinical environments, where training or deploying heavy transformer-based models or large foundation models may not be feasible. Accordingly, our evaluation centers on general-purpose convolutional neural network-based classifiers, including DLAD-10, an early commercial model, to assess whether large-scale public datasets alone can enable clinically acceptable performance without reliance on computationally intensive architectures.

### 2.4. UQ Using Monte Carlo (MC) Dropout

We employed MC dropout [34] to estimate model uncertainty by enabling stochastic dropout during inference, thereby generating multiple predictions for each input. This approach captures both aleatoric uncertainty (inherent data noise) and epistemic uncertainty (model or data limitations). Multiple forward passes were performed, and their predictions were aggregated to compute predictive entropy (PE) [35]—reflecting total uncertainty—and variance of predictions (VP) [36]—a direct measure of epistemic uncertainty. For robust estimation, we used a dropout rate of 0.4 and 20 stochastic passes, balancing computational efficiency with reliable metrics. The final class prediction was selected by averaging the probabilities across all passes. The dropout rate follows the standard configuration in previous studies [30,37], and the number of stochastic passes was chosen based on sensitivity analysis on the test set (see Appendix C for details).

### 2.5. Statistical Analysis

Diagnostic performance was evaluated using precision, sensitivity, specificity, F1-score, and area under the receiver operating characteristic curve (AUROC). These metrics provided a comprehensive assessment of the ability of the model to accurately classify disease states in chest radiographs, offering insights into discriminative capability and clinical utility. A 10-fold cross-validation was conducted, with performance reported as the average values across all folds, and 95% confidence intervals were calculated to assess variability and reliability. Stratified splitting was applied to ensure that each fold was representative of all target diseases, and multiple random seeds were evaluated to prevent folds with zero samples in low-prevalence classes; a seed was ultimately selected that preserved at least two samples per disease in every fold.

Class-specific decision thresholds (provided in Table 3 and Table 4) were optimized on the validation set by selecting operating points that favor sensitivity while avoiding excessive loss of specificity. Consistent with the clinical imperative to minimize false-negative findings—particularly for conditions such as tuberculosis, pneumonia, or pneumothorax, where missed diagnoses carry substantial risk—sensitivity was modestly prioritized. This approach acknowledges that while false positives necessitate additional confirmatory evaluation, they present significantly lower clinical risk than delayed diagnosis and treatment.

## 3. Results

### 3.1. Model Performance on Internal Validation Sets

**Figure 1:**
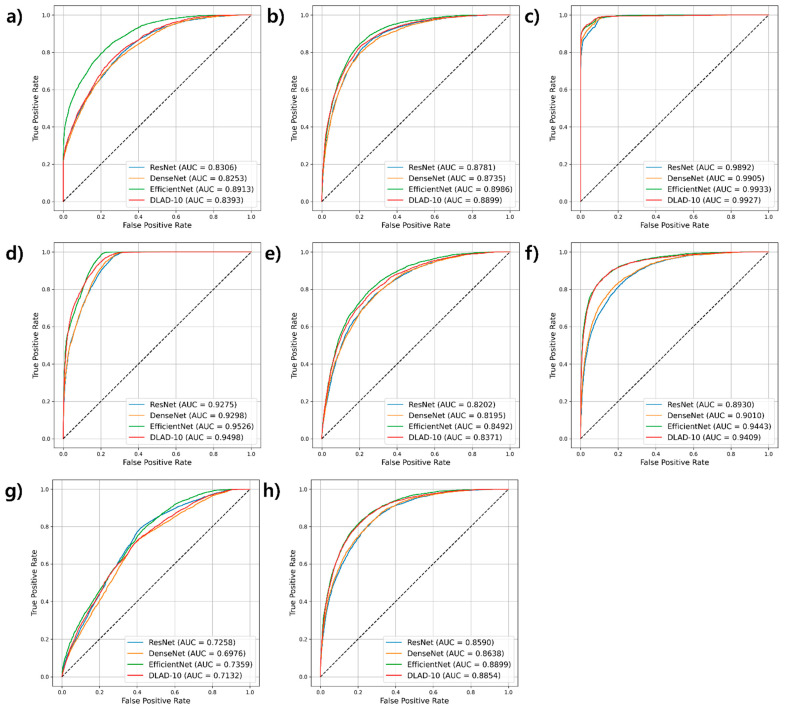
Comparison of AUROCs for deep learning models across different diseases using the development dataset. (**a**) Pneumonia, (**b**) Pleural effusion, (**c**) Tuberculosis, (**d**) Mass, (**e**) Consolidation, (**f**) Pneumothorax, (**g**) Other diseases, and (**h**) No finding (AUROC: area under the receiver operating characteristic curve).

Figure 1 and Table 3 summarize the performance of four deep learning models—ResNet, DenseNet, EfficientNet, and DLAD-10—on the internal validation sets. EfficientNet achieved the highest overall performance, with an average AUROC (0.8944) among all models. This model consistently outperformed the other models in sensitivity (0.81) and F1-score (0.61), reflecting its ability to accurately detect diseases while maintaining a balance between precision and recall (Table 3). DLAD-10 closely followed with an average AUROC of 0.8810, and although EfficientNet had a slight edge overall, disease-specific analysis revealed instances where DLAD-10 outperformed EfficientNet in certain categories, indicating that the relative advantage can vary by disease. In contrast, ResNet and DenseNet had slightly lower average AUROCs (0.8654 and 0.8626, respectively), with DenseNet exhibiting a marginally better F1-score than ResNet.

### 3.2. Model Performance on External Validation Set

**Figure 2:**
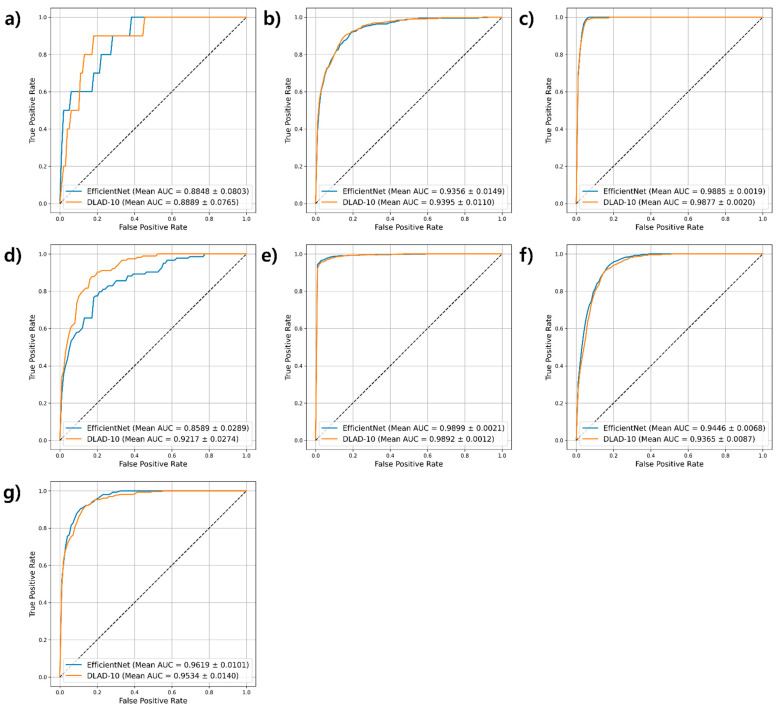
Comparison of AUROCs for deep learning models across different diseases using the external validation dataset. (**a**) Pneumonia, (**b**) Pleural effusion, (**c**) Tuberculosis, (**d**) Mass, (**e**) Pneumothorax, (**f**) Other diseases, and (**g**) No finding (AUROC: area under the receiver operating characteristic curve).

For the external validation set, analysis focused on EfficientNet, which demonstrated the best performance using the development dataset. As shown in Figure 2 and Table 4, EfficientNet demonstrated comparable or superior performance to DLAD-10 across most disease categories, consistent with its performance on the development dataset. Fine-tuning with a portion of the external validation dataset consistently improved the results of both models across most target diseases. However, performance variations were observed between internal and external datasets. Pneumonia, tuberculosis, and mass showed higher AUROC scores internally, whereas the external dataset yielded better performance for the remaining diseases. These differences likely stemmed from the smaller number of samples for certain diseases in the external dataset, affecting generalizability. Overall, EfficientNet maintained strong diagnostic accuracy on the external validation set, demonstrating adaptability to diverse clinical scenarios.

### 3.3. Impact of Training Data Scale and Diversity on Model Effectiveness

#### 3.3.1. Diagnostic Performance Across Data Scales

To evaluate the impact of training data scale, we incrementally increased the dataset size (20%, 40%, 60%, and 100% of 40,000 CXR images per disease) while maintaining proportional representation from source datasets. Experiments were conducted under controlled conditions using a development dataset, with MC dropout applied during inference to estimate uncertainty.

As presented in Table 5, model performance consistently improved with larger datasets: the mean AUROC rose from 0.8680 (20%) to 0.8937 (100%), and precision (0.47–0.51), sensitivity (0.80–0.82), specificity (0.78–0.81), and F1-score (0.57–0.61) all increased. Disease-specific results revealed significant gains for pneumothorax (0.9115–0.9437), pleural effusion (0.8657–0.8978), mass (0.9416–0.9520), and pneumonia (0.8414–0.8905), underscoring the benefit of expanded training data. In contrast, tuberculosis showed a slight AUROC decline (0.9937–0.9926). Given the relatively small number of unique tuberculosis cases (3929 studies) and the substantial reliance on duplicated samples to reach the target dataset size, this trend may reflect the limited diversity of underlying image features. However, other factors—including label variability across datasets or differences in acquisition protocols—may also contribute, and therefore this behavior should be interpreted with caution. Consolidation and the “Other” disease category also improved, although less dramatically. Overall, these findings confirm that larger, more diverse training datasets enhance model performance for well-represented diseases but underscore the need for targeted strategies when addressing rare or underrepresented conditions, including tuberculosis.

#### 3.3.2. Diagnostic Performance Across Data Diversity

We trained models using two configurations to assess the impact of data diversity. In the single-source setup, each disease category comprised 5000 samples drawn from one public dataset (Chest X-Ray-14, CheXpert, MIMIC-CXR, or PadChest), with duplicates added as needed. In the multiple-source setup, 1250 samples per disease were selected from each dataset to form a combined set of 5000 images, thereby increasing diversity. An independent test dataset (Table 2) ensured consistent evaluation under uniform conditions. All models were trained under uniform conditions.

Table 6 indicates that diagnostic performance generally improved with the multiple-source dataset: mean AUROC increased from 0.7061 (Chest X-Ray-14-only) and 0.7523 (MIMIC-CXR-only) to 0.7708, with sensitivity and specificity rising to 0.63 and 0.75, respectively. However, the degree of improvement varied across diseases and metrics. For example, the AUROC for mass increased substantially from 0.6765 to 0.8850, and similar trends were observed for pneumonia and pleural effusion, whereas the “Other” disease category exhibited only modest gains (AUROC from 0.5324 to 0.5669). In some cases, single-source datasets outperformed the multiple-source configuration for specific metrics (e.g., MIMIC-CXR-only achieved a higher specificity for pneumonia: 0.72 vs. 0.65).

Tuberculosis exhibited unique behavior because its samples were exclusively derived from the PadChest dataset, rendering its data effectively single-source even in the multiple-source configuration. The AUROC improved from 0.3891 (PadChest-only) to 0.6440 with the multiple-source dataset, while precision and specificity showed inconsistent trends. This discrepancy likely reflects the influence of diverse non-tuberculosis samples on overall model calibration, while limited variability in tuberculosis-specific data constrained further improvements. Overall, these results demonstrate that data diversity enhances performance for common thoracic conditions, while highlighting the need for targeted data collection strategies to improve generalization for rare or heterogeneous diseases.

#### 3.3.3. Uncertainty and Data Scale Relationship

As presented in Table 7, a descriptive analysis of PE and VP across increasing training data scales (20%, 40%, 60%, and 100%) reveals that VP generally decreases or stabilizes across all disease categories. Pleural effusion, consolidation, other diseases, and no finding exhibited clear decreases followed by stable low VP values (e.g., consolidation: 0.0007 at 20% to 0.0004 from 40% onward). Mass showed the largest reduction (0.0009 to 0.0005 and then to 0.0004), while pneumothorax remained unchanged through 60% (0.0004) and then decreased at 100% (0.0003). Pneumonia displayed a mild non-monotonic pattern, and tuberculosis remained constant at 0.0001 across all data scales.

Conversely, PE varied substantially by class and did not follow a monotonic trend. Most diseases showed a noticeable reduction between 20% and 40% data—such as pneumonia (1.9592 to 1.616) and consolidation (2.2179 to 1.893)—but increased again at 60% or 100%. Additionally, pneumothorax decreased sharply at 40% and partially rebounded at higher scales, while tuberculosis demonstrated the largest fluctuation relative to its scale (0.1858 → 0.0812 → 0.3547 → 0.2172) despite stable VP. The “Other” disease category exhibited a distinct pattern, with PE increasing from 20% to 60% (1.0883 to 1.3439) but decreasing sharply at 100% (0.6445). This substantial drop is attributable to the broad heterogeneity of the class: with limited data, the model is highly uncertain because it has insufficient exposure to the diverse subpatterns within this category. As data volume increases, the model encounters a wider and more representative range of these patterns, reducing epistemic uncertainty and yielding more stable calibration across this heterogeneous class.

Overall, these results demonstrate that scaling the training data reliably reduces VP across diseases, whereas PE is driven predominantly by class-specific factors including heterogeneity, rarity, and label consistency. Therefore, total predictive uncertainty does not necessarily decrease with more data, even when epistemic uncertainty becomes stable.

## 4. Discussion

Our study demonstrates that large-scale, diverse public CXR datasets with noisy labels can effectively train deep learning models for disease classification, overcoming limitations of imperfect data through scalability and diversity. However, rare and underrepresented conditions remain challenging, underscoring the need for targeted strategies, including higher-quality data collection and advanced model architectures tailored to their characteristics.

A key contribution of our study is the integration of UQ to analyze how predictive certainty evolves with increasing data sizes and across disease categories. As shown in our results, VP consistently decreased or stabilized as the training data expanded from 20% to 40%, 60%, and 100%, indicating improved parameter certainty under more balanced data regimes. In contrast, PE displayed heterogeneous and frequently non-monotonic trajectories, demonstrating that total predictive uncertainty is shaped not only by dataset size but also by inherent class characteristics, including heterogeneity, annotation quality, and imaging variability. For example, pneumonia, pleural effusion, and consolidation showed an initial reduction in PE but exhibited secondary increases at larger data scales, suggesting the presence of persistent aleatoric uncertainty that cannot be resolved solely by increasing training samples. Pneumothorax exhibited a similar pattern, with PE decreasing substantially at 40% data and subsequently increasing at higher scales despite stable VP.

The tuberculosis category exhibited a distinctive uncertainty pattern: VP remained constant at 0.0001 across all data scales, whereas PE fluctuated substantially. This behavior likely reflects the limited diversity of tuberculosis images in the original datasets and the extensive oversampling required to match the training volume of other diseases. Because the model repeatedly encounters a narrow set of visual patterns during training, epistemic variability remains minimal, resulting in stable VP values. However, PE remains sensitive to residual data-level ambiguities—such as heterogeneous imaging conditions, variations in disease presentation, and inconsistencies in text-derived labels—leading to the observed fluctuations. This contrast highlights how VP and PE capture different aspects of model uncertainty: while VP reflects stability in parameter estimates under repeated sampling, PE is influenced more strongly by intrinsic variability within the underlying data distribution.

These class-specific uncertainty profiles can be further interpreted by examining the dataset composition and adjusted training counts presented in Table 1 and Table 2, respectively. Several rare diseases—most notably tuberculosis—were severely underrepresented in the original datasets (~3.9 k of ~1.08 M) and required extensive oversampling and augmentation to balance the training distribution. Under these conditions, VP naturally stabilized due to repeated sampling of similar examples, whereas PE remained sensitive to unresolved label inconsistencies and limited intra-class diversity. Mass and pneumothorax, both of which rely heavily on NLP-derived labels, also showed PE fluctuations consistent with known variability in automated labeling pipelines and cross-dataset domain heterogeneity. Conversely, the substantial PE reduction observed for the “Other” diseases category at 100% data reflects the stabilizing effect of its exceptionally large and diverse original sample pool; only when exposed to the full dataset could the model approximate the underlying distribution sufficiently to reduce total uncertainty.

Direct comparisons with previous studies are challenging because of differences in dataset composition, labeling quality, and model architectures. Many previous studies relied on private or mixed private-public datasets, whereas our approach used only public datasets, enhancing accessibility but introducing variability and noise. Moreover, our study incorporated UQ to assess predictive certainty, a dimension frequently overlooked in similar research. Tang et al. [38] demonstrated robust binary classification using well-established models, while our study extends this approach to multiclass classification using an even larger dataset and highlights the benefits of fine-tuning for domain adaptation. Wu et al. [6] developed a custom model to classify 72 thoracic diseases from 353,818 CXR images from the NIH and MIMIC datasets using NLP-based automated labeling, achieving a mean AUROC of 0.807—comparable to a third-year radiology resident. Similarly, Cid et al. [7] utilized 1,896,034 images from three UK hospitals to classify 37 diseases, attaining a mean AUROC of 0.864, underscoring the impact of dataset scale on diagnostic accuracy. More recently, Seah et al. [8] trained EfficientNet on 821,681 images from five public datasets (including NIH Chest X-ray-14 and MIMIC) with radiologist-labeled high-quality data, achieving an outstanding mean AUROC of 0.957. A key distinction of our study is the methodological focus imposed by our open-source approach. While these large-scale studies [7,8] utilized private or highly curated institutional data and concentrated on maximizing accuracy within custom, complex taxonomies, our use of entirely public datasets necessitates and introduces multi-dataset harmonization and a focus on cross-dataset generalization. This methodological foundation, combined with our uncertainty-aware analysis, demonstrates high diagnostic performance using fully open-source resources across clinically significant multiclass conditions, broadening the applicability of automated diagnostic solutions, particularly in resource-constrained settings.

This study had some limitations. First, the external validation dataset was derived from a single institution, and the uneven distribution of cases for different diseases may not reflect real-world prevalence, potentially limiting generalizability. Second, although we aggregated 17 public datasets, substantial upsampling was required for several rare disease classes to maintain controlled comparisons across categories. While all unique and duplicated sample counts are transparently reported, reliance on duplicated images may constrain feature diversity for these underrepresented classes. Third, the “Other” diseases category encompasses a broad and heterogeneous set of abnormalities, and although we provide detailed label mappings, such heterogeneity inevitably reduces class-specific interpretability compared with well-defined disease categories. Fourth, although labels were harmonized and cross-dataset duplicates were removed, residual domain shifts arising from differences in imaging protocols, patient demographics, and disease prevalence across the 17 public datasets may still affect model generalizability, particularly for conditions with inconsistent labeling strategies. Fifth, we focused on six thoracic conditions, restricting their applicability to various diseases. Sixth, we employed MC dropout as the sole UQ method, leaving alternative techniques unexplored. Finally, we utilized a subset of state-of-the-art deep learning models, which may not fully leverage more recent architectures, including Vision Transformers or large foundation models; while our findings on scalability and uncertainty are expected to generalize, evaluating these newer models remains an avenue for future investigation.

## 5. Conclusions

This study demonstrated the feasibility of leveraging public datasets and open-source deep learning models to achieve robust diagnostic performance in CXR analysis. Using data scale and diversity, our approach achieved results comparable to those obtained with proprietary datasets while providing a fully reproducible and accessible framework. Our uncertainty analysis further highlighted that, although epistemic uncertainty decreases with increasing data, total predictive uncertainty remains strongly influenced by class-specific characteristics, label quality, and inherent data heterogeneity. These findings underscore the need for more diverse and high-quality datasets—particularly for rare or underrepresented conditions—to further improve model reliability and stability. Despite these challenges, our study advances the democratization of AI tools for medical imaging by providing a scalable, uncertainty-aware framework that facilitates broader adoption in diverse and resource-constrained clinical settings.

## Figures and Tables

**Table 1:** Summary of publicly available CXR datasets used in the study (NLP: Natural Language Processing, NN: Neural Network).

Datasets	No. of Labels	Annotation Method	No. of CXRs								
			Pneumonia	Pleural Effusion	Tuberculosis	Mass	Consolidation	Pneumothorax	Other Diseases	No Finding	Total
NIH Chest X-ray-14 [9]	14	An NLP Tool	1431	13,317	0	11,207	4667	5302	36,251	60,361	112,120
CheXpert [10]	14	An NLP Tool	3738	79,713	0	0	12,170	16,257	144,789	15,892	224,316
MIMIC-CXR [11]	14	An NLP Tool	26,221	76,954	0	0	14,675	14,257	180,251	143,351	377,110
PadChest [15]	19	27% of reports were manually annotated and the rest using a supervised NN.	4138	5441	647	3034	1426	345	52,515	28,999	160,000
VinDr-CXR [17]	28	Radiologists	1229	1430	1006	1080	444	133	6809	10,606	100,000
RSNA Pneumonia Detection Challenge [18]	2	Radiologists	6012	0	0	0	0	0	0	20,672	26,684
SIIM-ACR Pneumothorax Segmentation [19]	2	Radiologists	0	0	0	0	0	2379	0	8296	10,675
JSRT [20]	2	Radiologists	0	0	0	154	0	0	93	0	247
Shenzhen Hospital CXR Set [21]	4	Radiologists	0	34	336	132	18	0	272	326	662
Montgomery County chest X-ray set (MC) [21]	2	Radiologists	0	0	58	0	0	0	0	80	138
BRAX [16]	14	An NLP Tool	264	532	0	0	1120	38	3612	14,782	40,967
COVID-19, Pneumonia and Normal Chest X-ray PA Dataset [22]	3	Radiologists	1525	0	0	0	0	0	0	1525	4575
Chest X-Ray Images (Pneumonia) [23]	2	Radiologists	3875	0	0	0	0	0	0	1341	5856
Tuberculosis (TB) Chest X-ray Database [24]	2	Radiologists	0	0	700	0	0	0	0	3500	4200
TBX11K [25]	5	Radiologists	0	0	800	0	0	0	3800	3800	11,200
Belarus Dataset [26]	1	Radiologists	0	0	304	0	0	0	0	0	304
Chest X-rays tuberculosis from India [27]	2	Radiologists	0	0	78	0	0	0	0	77	156
Total	−	−	48,433	177,421	3929	15,607	34,520	38,711	428,392	313,608	1,079,210

**Table 2:** Data distribution per target disease for the training, validation, and testing sets of the deep learning models.

Datasets	Development Dataset				External Validation Dataset
	No. of Total CXR Images	Training	Validation	Testing	No. of Total CXR Images
Pneumonia	48,433	40,000	9237	2421	10
Pleural Effusion	177,421	40,000	9721	2990	422
Tuberculosis	3929	40,000	7820	1237	318
Mass	15,607	40,000	9763	2945	69
Consolidation	34,520	40,000	9884	2988	0
Pneumothorax	38,711	40,000	9982	2998	1312
Other diseases	428,299	68,525	12,277	3024	843
No finding	313,701	39,999	9807	2936	240

**Table 3:** Performance of deep learning models and selected thresholds for each disease using the development dataset.

Target Diseases	Threshold				ResNet	DenseNet	EfficientNet	DLAD-10
	ResNet	DenseNet	EfficientNet	DLAD-10				
Pneumonia	0.28	0.24	0.255	0.20	(0.31, 0.74, 0.74, 0.43)	(0.30, 0.74, 0.74, 0.43)	(0.37, 0.80, 0.79, 0.51)	(0.33, 0.74, 0.76, 0.46)
Pleural Effusion	0.25	0.145	0.215	0.16	(0.45, 0.80, 0.80, 0.57)	(0.45, 0.78, 0.81, 0.57)	(0.47, 0.83, 0.81, 0.60)	(0.46, 0.81, 0.81, 0.59)
Tuberculosis	0.50	0.50	0.50	0.50	(0.98, 0.73, 1.0, 0.84)	(0.98, 0.81, 1.00, 0.89)	(0.98, 0.89, 1.00, 0.93)	(0.99, 0.86, 1.00, 0.92)
Mass	0.35	0.29	0.10	0.29	(0.51, 0.82, 0.84, 0.63)	(0.51, 0.84, 0.84, 0.64)	(0.57, 0.87, 0.87, 0.69)	(0.56, 0.86, 0.87, 0.68)
Consolidation	0.18	0.19	0.20	0.20	(0.36, 0.75, 0.74, 0.49)	(0.36, 0.74, 0.74, 0.48)	(0.40, 0.76, 0.77, 0.52)	(0.38, 0.76, 0.76, 0.51)
Pneumothorax	0.18	0.15	0.18	0.18	(0.45, 0.81, 0.81, 0.58)	(0.47, 0.81, 0.82, 0.60)	(0.58, 0.86, 0.88, 0.70)	(0.59, 0.86, 0.88, 0.70)
Other diseases	0.35	0.30	0.38	0.38	(0.29, 0.66, 0.67, 0.40)	(0.27, 0.65, 0.65, 0.38)	(0.28, 0.65, 0.67, 0.39)	(0.27, 0.64, 0.66, 0.38)
No finding	0.23	0.24	0.30	0.24	(0.40, 0.77, 0.78, 0.53)	(0.40, 0.77, 0.77, 0.53)	(0.45, 0.80, 0.81, 0.58)	(0.44, 0.80, 0.80, 0.57)

Data in parentheses indicate precision, sensitivity, specificity, and F1-score.

**Table 4:** Performance of deep learning models and selected thresholds for each disease using the external validation dataset.

Target Diseases	With Fine-Tuning				Without Fine-Tuning	
	Thresholds		EfficientNet	DLAD-10	EfficientNet	DLAD-10
	EfficientNet	DLAD-10				
Pneumonia	0.255	0.20	(0.00, 0.00, 1.00, 0.00)	(0.00, 0.00, 1.00, 0.00)	(0.01, 0.40, 0.76, 0.01)	(0.01, 0.70, 0.68, 0.01)
Pleural Effusion	0.215	0.16	(0.59, 0.78, 0.91, 0.67)	(0.55, 0.83, 0.89, 0.66)	(0.30, 0.70, 0.74, 0.42)	(0.31, 0.74, 0.73, 0.44)
Tuberculosis	0.5	0.50	(0.82, 0.88, 0.98, 0.84)	(0.84, 0.80, 0.98, 0.80)	(0.15, 0.08, 0.95, 0.10)	(0.23, 0.08, 0.97, 0.12)
Mass	0.1	0.29	(0.23, 0.48, 0.96, 0.31)	(0.40, 0.19, 0.99, 0.21)	(0.04, 0.41, 0.74, 0.07)	(0.05, 0.39, 0.84, 0.09)
Consolidation	–	–	–	–	–	–
Pneumothorax	0.18	0.18	(0.94, 0.97, 0.96, 0.96)	(0.90, 0.98, 0.92, 0.94)	(0.88, 0.84, 0.91, 0.86)	(0.88, 0.86, 0.91, 0.87)
Other diseases	0.38	0.38	(0.73, 0.87, 0.87, 0.79)	(0.73, 0.84, 0.88, 0.78)	(0.31, 0.68, 0.42, 0.43)	(0.32, 0.63, 0.48, 0.42)
No finding	0.3	0.24	(0.61, 0.77, 0.96, 0.68)	(0.60, 0.73, 0.96, 0.65)	(0.24, 0.87, 0.76, 0.37)	(0.23, 0.92, 0.74, 0.37)

Data in parentheses indicate precision, sensitivity, specificity, and F1-Score.

**Table 5:** Diagnostic performance for each target condition across different training data scales using the external validation dataset (*N*: number of samples used for training, AUROC: area under the receiver operating characteristic curve).

Target Diseases	Case−1			Case−2			Case−3			Case−4		
	*N*	Performance	AUROC	*N*	Performance	AUROC	*N*	Performance	AUROC	*N*	Performance	AUROC
Pneumonia	7913	(0.32, 0.75, 0.75, 0.45)	0.8414	16,077	(0.34, 0.76, 0.79, 0.48)	0.8689	24,017	(0.37, 0.79, 0.79, 0.50)	0.8819	40,000	(0.37, 0.79, 0.80, 0.51)	0.8905
Pleural Effusion	8005	(0.42, 0.78, 0.78, 0.55)	0.8657	15,908	(0.45, 0.81, 0.81, 0.58)	0.8881	24,031	(0.46, 0.81, 0.82, 0.59)	0.8900	40,000	(0.47, 0.81, 0.82, 0.57)	0.8978
Tuberculosis	7977	(0.97, 0.99, 0.87, 0.92)	0.9937	16,071	(0.96, 0.99, 0.90, 0.93)	0.9948	24,029	(0.96, 0.99, 0.90, 0.93)	0.9930	40,000	(0.98, 0.99, 0.88, 0.93)	0.9926
Mass	7964	(0.54, 0.86, 0.86, 0.66)	0.9416	15,993	(0.55, 0.87, 0.86, 0.67)	0.9494	24,039	(0.58, 0.88, 0.88, 0.70)	0.9580	40,000	(0.57, 0.87, 0.87, 0.69)	0.9520
Consolidation	8090	(0.36, 0.74, 0.73, 0.48)	0.8139	16,109	(0.38, 0.76, 0.76, 0.51)	0.8365	24,029	(0.39, 0.77, 0.77, 0.52)	0.8488	40,000	(0.40, 0.77, 0.76, 0.52)	0.8485
Pneumothorax	8097	(0.49, 0.83, 0.82, 0.61)	0.9115	15,958	(0.53, 0.85, 0.84, 0.65)	0.9267	23,928	(0.54, 0.85, 0.86, 0.67)	0.9375	40,000	(0.58, 0.88, 0.86, 0.70)	0.9437
Other diseases	13,777	(0.27, 0.63, 0.67, 0.38)	0.7141	27,447	(0.26, 0.64, 0.64, 0.37)	0.7098	41,072	(0.27, 0.64, 0.65, 0.38)	0.7186	68,525	(0.28, 0.67, 0.65, 0.39)	0.7354
No finding	7933	(0.41, 0.78, 0.79, 0.54)	0.8623	15,972	(0.43, 0.80, 0.79, 0.56)	0.8745	23,995	(0.43, 0.79, 0.80, 0.56)	0.8817	39,999	(0.44, 0.80, 0.80, 0.57)	0.8891

Data in parentheses indicate precision, sensitivity, specificity, and F1-score.

**Table 6:** Diagnostic performance for each target condition across datasets with varying diversity (AUROC: area under the receiver operating characteristic curve).

Target Diseases	NIH Chest X-Ray-14		MIMIC-CXR		PadChest		CheXpert		Multiple-Source	
	Performance	AUROC	Performance	AUROC	Performance	AUROC	Performance	AUROC	Performance	AUROC
Pneumonia	(0.21, 0.62, 0.63, 0.31)	0.6695	(0.21, 0.48, 0.72, 0.29)	0.6593	(0.13, 0.48, 0.51, 0.21)	0.4829	(0.21, 0.62, 0.65, 0.32)	0.6723	(0.22, 0.66, 0.65, 0.33)	0.7184
Pleural Effusion	(0.32, 0.72, 0.69, 0.44)	0.7915	(0.40, 0.78, 0.77, 0.53)	0.8479	(0.19, 0.40, 0.66, 0.26)	0.5511	(0.37, 0.75, 0.75, 0.50)	0.8209	(0.38, 0.83, 0.73, 0.52)	0.8574
Tuberculosis	–	–	–	–	(0.05, 0.59, 0.26, 0.10)	0.3891	–	–	(0.05, 0.01, 0.99, 0.01)	0.6440
Mass	(0.23, 0.62, 0.60, 0.34)	0.6765	–	–	(0.08, 0.14, 0.68, 0.10)	0.3969	–	–	(0.41, 0.79, 0.78, 0.54)	0.8850
Consolidation	(0.24, 0.63, 0.60, 0.34)	0.6656	(0.27, 0.66, 0.64, 0.38)	0.7059	(0.15, 0.40, 0.56, 0.22)	0.4804	(0.27, 0.67, 0.64, 0.38)	0.7131	(0.31, 0.70, 0.70, 0.43)	0.7636
Pneumothorax	(0.39, 0.75, 0.77, 0.52)	0.8393	(0.44, 0.77, 0.80, 0.56)	0.8625	(0.15, 0.28, 0.69, 0.20)	0.4997	(0.44, 0.76, 0.80, 0.56)	0.8514	(0.46, 0.83, 0.81, 0.60)	0.9002
Other diseases	(0.17, 0.52, 0.51, 0.26)	0.5324	(0.22, 0.60, 0.57, 0.32)	0.6214	(0.16, 0.31, 0.66, 0.21)	0.4560	(0.18, 0.52, 0.51, 0.26)	0.5538	(0.20, 0.51, 0.59, 0.29)	0.5669
No finding	(0.30, 0.72, 0.67, 0.42)	0.7680	(0.36, 0.74, 0.74, 0.49)	0.8167	(0.16, 0.42, 0.56, 0.23)	0.4943	(0.31, 0.70, 0.70, 0.43)	0.7687	(0.37, 0.76, 0.75, 0.50)	0.8312

The models trained on Chest X-ray-14, MIMIC-CXR, and CheXpert lacked tuberculosis classification capability, whereas those trained on MIMIC-CXR and CheXpert lacked mass classification because of the absence of corresponding samples in these datasets. Data in parentheses indicate precision, sensitivity, specificity, and F1-score.

**Table 7:** Predictive entropy (PE) and variance of predictions (VP) for each target condition across different training data scales using the external validation dataset (*N*: number of samples used for training).

Target Diseases	Case−1			Case−2			Case−3			Case−4		
	*N*	PE	VP	*N*	PE	VP	*N*	PE	VP	*N*	PE	VP
Pneumonia	7913	1.9592	0.0007	16,077	1.616	0.0004	24,017	1.9135	0.0005	40,000	1.772	0.0004
Pleural Effusion	8005	1.8092	0.0006	15,908	1.506	0.0005	24,031	1.5154	0.0004	40,000	1.7032	0.0004
Tuberculosis	7977	0.1858	0.0001	16,071	0.0812	0.0001	24,029	0.3547	0.0001	40,000	0.2172	0.0001
Mass	7964	2.1449	0.0009	15,993	2.0338	0.0005	24,039	1.9871	0.0004	40,000	2.228	0.0004
Consolidation	8090	2.2179	0.0007	16,109	1.893	0.0004	24,029	1.6253	0.0004	40,000	1.9582	0.0004
Pneumothorax	8097	2.2859	0.0004	15,958	1.8744	0.0004	23,928	2.0114	0.0004	40,000	2.0566	0.0003
Other diseases	13,777	1.0883	0.0009	27,447	1.1631	0.0006	41,072	1.3439	0.0005	68,525	0.6445	0.0005
No finding	7933	1.4209	0.0007	15,972	0.6132	0.0005	23,995	1.446	0.0005	39,999	0.7514	0.0005

## Data Availability

Public datasets used in this study are described in the text and tables and are available from the corresponding author upon request. The in-house dataset supporting these findings is maintained at Keimyung University Dongsan Hospital. Due to licensing restrictions, these data are not publicly available; however, access may be granted upon reasonable request and subject to approval by Keimyung University Dongsan Hospital.

## Abbreviations

The following abbreviations are used in this manuscript:

AI	Artificial Intelligence
AUROC	Area Under the Receiver Operating Characteristics Curve
CXR	Chest X-ray
DLAD-10	Deep Learning-based Algorithm for Detecting 10 Abnormalities
MC	Monte Carlo
MIMIC-CXR	Medical Information Mart for Intensive Care—Chest X-ray
NIH	National Institute of Health
NLP	Natural Language Processing
PA	Posterior–Anterior
PE	Predictive Entropy
TB	Tuberculosis
UQ	Uncertainty quantification
VP	Variance of Predictions

## Appendix A

We implemented four deep learning architectures for CXR classification—ResNet-50, DenseNet-121, EfficientNet-B5 and DLAD-10—chosen for their demonstrated effectiveness in natural image tasks and established performance in chest radiograph analysis. The original classification layer of each model was replaced with a linear layer corresponding to the dataset labels. The models were trained as multi-label classifiers, producing independent sigmoid outputs for each class, using binary cross-entropy with logit loss. This loss is suitable for multi-label classification, where each class is treated independently. The model parameters were optimized using the Adam optimizer. Class-specific decision thresholds were determined by evaluating performance metrics across a range of thresholds (0.0–1.0) on the validation set. Thresholds were selected at points where sensitivity and specificity were approximately balanced, with slight prioritization of sensitivity to minimize false negatives. The resulting disease-specific thresholds were subsequently applied unchanged to the test set to ensure unbiased evaluation.

Before input into the models, each input CXR image was subjected to lung segmentation using U-Net [39] architecture trained specifically for this study. The model was initialized with random weights and trained on a combined dataset of 7951 images from the Mendeley Chest X-ray Lung Segmentation dataset [40] and the MIMIC-CXR segmentation subset [41], split into 70–20–10 training, validation, and test sets. During segmentation model training, data augmentation included horizontal flipping, color jittering (brightness, contrast, and saturation ± 0.5; hue ± 0.3), conversion to grayscale, and resizing to 512 × 512 pixels. The model achieved a Dice coefficient of 0.9251 on an independent test set (*n* = 796), indicating accurate delineation of lung fields (see Table A1). The resulting segmentation masks were used to crop each image to the lung region, removing non-informative background areas. The cropped images were subsequently resized to 448 × 448 pixels.

For classification model training, online data augmentation was applied using PyTorch 2.3.0, deliberately employing milder transformations, random rotations up to ±20° and color jittering (brightness, contrast, and saturation ± 0.2, hue ± 0.1) than those used for segmentation, ensuring preservation of critical anatomical detail while still promoting generalization. To reduce overfitting, each image was uniquely augmented at every iteration. Subsequently, input images were resized and normalized using the standard ImageNet mean and standard deviation to ensure compatibility with the ImageNet-pretrained backbone used for initialization, preventing a distribution mismatch that could degrade performance. While ImageNet statistics originate from natural RGB images, their use in chest X-rays is widely adopted and empirically validated when using pretrained models. Chest X-rays are inherently single-channel; they are duplicated across three channels to match the input requirements of ImageNet-pretrained models. Therefore, normalization primarily standardizes the input range and stabilizes gradients without distorting diagnostic structures. Furthermore, widely used medical imaging benchmarks—including CheXpert [10], MIMIC-CXR [11], RSNA [18], and SIIM-ACR pneumothorax [19] datasets—commonly adopt ImageNet normalization with pretrained backbones, demonstrating its practicality and suitability for radiography tasks. The models’ training was configured for up to 30 epochs with early stopping using a batch size of 32. The initial learning rate started at 0.001 and was reduced upon validation loss plateau. Fine-tuning employed the same parameters with a reduced learning rate of 0.0001.

**Table A1:** Performance metrics of the U-Net architecture used for lung segmentation (preprocessing) on the independent test set (*n* = 796).

Metrics	Value
Dice Coefficient	0.9251
IoU	0.8573
Precision	0.9396
Recall	0.9073

## Appendix B

To ensure consistent and high-quality annotations across the 17 aggregated CXR datasets, several harmonization steps were applied. Only frontal-view CXRs (AP/PA) were included to maintain clinical consistency. Labels with low confidence or uncertainty (e.g., CheXpert’s “Uncertain”) were excluded from the training set to maintain label reliability. Annotation protocols varied across datasets in both naming conventions and label granularity. To ensure consistency, labels referring to related abnormalities (e.g., “mass” and “nodule”) were consolidated into a single Mass category. Findings outside the primary target disease set, including cardiomegaly, atelectasis, and fibrosis, were grouped into an “Other” disease category to maintain a unified label structure. PadChest [15] provides 174 radiographic findings extracted via NLP from full radiology reports; for this dataset, a target disease was marked positive whenever its corresponding condition appeared within PadChest’s annotated label list. Duplicate studies across datasets were identified and removed to prevent overlap between the training and evaluation sets, particularly for large public datasets, including CheXpert [10] and MIMIC-CXR [11].

To mitigate class imbalance, images from underrepresented classes were duplicated in the training set. Each image was copied without alteration to preserve its original characteristics. The distribution of original images, duplicates added, and total images after duplication is summarized in Table A2.

All harmonization rules and label mappings are mentioned in Table A3, enabling full reproducibility and transparency in dataset construction.

**Table A2:** Class distribution before and after image duplication: (**a**) Training Set, (**b**) Validation Set, and (**c**) Test Set.

**(a)**			
**Class**	**Unique Images**	**Duplicates Added**	**Total Images**
Pneumonia	35,987	4013	40,000
Pleural Effusion	33,360	6640	40,000
Tuberculosis	2301	37,699	40,000
Mass	12,156	27,844	40,000
Consolidation	31,002	8998	40,000
Pneumothorax	35,160	4840	40,000
Other diseases	67,576	949	68,525
No finding	39,999	0	39,999
**(b)**			
**Class**	**Unique Images**	**Duplicates added**	**Total Images**
Pneumonia	1479	7758	9237
Pleural Effusion	2996	6725	9721
Tuberculosis	391	7429	7820
Mass	506	9257	9763
Consolidation	530	9354	9884
Pneumothorax	553	9429	9982
Other diseases	1420	10,857	12,277
No finding	9807	0	9807
**(c)**			
**Class**	**Unique Images**	**Duplicates added**	**Total Images**
Pneumonia	2421	0	2421
Pleural Effusion	2990	0	2990
Tuberculosis	1237	0	1237
Mass	2945	0	2945
Consolidation	2988	0	2988
Pneumothorax	2998	0	2998
Other diseases	3024	0	3024
No finding	2936	0	2936

**Table A3:** Label Mapping Across Datasets.

Dataset	Original Dataset Label	Harmonized Label	Notes
NIH Chest X-ray-14 [9]	Atelectasis	Other	Not part of target diseases
	Cardiomegaly	Other	Not part of target diseases
	Effusion	Pleural Effusion	Retained as-is
	Infiltration	Other	Not part of target diseases
	Mass	Mass	Combined for consistency
	Nodule	Mass	Combined for consistency
	Pneumonia	Pneumonia	Retained as-is
CheXpert [10], MIMIC-CXR [11], BRAX [16]	Enlarged Cardiomediastinum	Other	Not part of target diseases
	Cardiomegaly	Other	Not part of target diseases
	Lung Lesion	Other	Not part of target diseases
	Lung Opacity	Other	Not part of target diseases
	Edema	Other	Not part of target diseases
	Consolidation	Consolidation	Retained as-is
	Pneumonia	Pneumonia	Retained as-is
	Atelectasis	Other	Not part of target diseases
	Pneumothorax	Pneumothorax	Retained as-is
	Pleural Effusion	Pleural Effusion	Retained as-is
	Pleural Other	Other	Not part of target diseases
	Fracture	Other	Not part of target diseases
	Support Devices	Support Devices	Retained as-is
PadChest [15] *	effusion	Pleural Effusion	Target disease assigned positive if present in PadChest label list
	nodule	Mass	
	mass	Nodule	
	consolidation	Consolidation	
	pneumonia	Pneumonia	
	tuberculosis	Tuberculosis	
VinDr-CXR [17]	Aortic enlargement	Other	Not part of target diseases
	Atelectasis	Other	Not part of target diseases
	Cardiomegaly	Other	Not part of target diseases
	Calcification	Other	Not part of target diseases
	Clavicle fracture	Other	Not part of target diseases
	Consolidation	Consolidation	Retained as-is
	Edema	Other	Not part of target diseases
	Emphysema	Other	Not part of target diseases
	Enlarged PA	Other	Not part of target diseases
	Interstitial lung disease (ILD)	Other	Not part of target diseases
	Infiltration	Other	Not part of target diseases
	Lung cavity	Other	Not part of target diseases
	Lung cyst	Other	Not part of target diseases
	Lung opacity	Other	Not part of target diseases
	Mediastinal shift	Other	Not part of target diseases
	Nodule/Mass	Mass	Renamed for consistency
	Pulmonary fibrosis	Other	Not part of target diseases
	Pneumothorax	Pneumothorax	Retained as-is
	Pleural thickening	Other	Not part of target diseases
	Pleural effusion	Pleural Effusion	Retained as-is
	Rib fracture	Other	Not part of target diseases
	Other lesion	Other	Not part of target diseases
	Lung tumor	Mass	Combined for consistency
	Pneumonia	Pneuonia	Retained as-is
	Tuberculosis	Tuberculosis	Retained as-is
	Other diseases	Other	Not part of target diseases
	Chronic obstructive pulmonary disease (COPD)	Other	Not part of target diseases
RSNA Pneumonia Detection Challenge [18]	Pneumonia	Pneumonia	Retained as-is
SIIM-ACR Pneumothorax Segmentation [19]	Pneummothorax	Pneumothorax	Retained as-is
JSRT [20]	Lung Nodule	Mass	Retained as-is
Shenzhen Hospital CXR Set [21] **	Tuberculosis	Tuberculosis	Retained as-is
	Effusion	Pleural Effusion	Extracted from ‘findings’
	Mass	Mass	Extracted from ‘findings’
	Consolidation	Consolidation	Extracted from ‘findings’
	Pleural thickening	Other	Not part of target diseases
	Fibrous lesions	Other	Not part of target diseases
Montgomery County chest X-ray set (MC) [21]	Tuberculosis	Tuberculosis	Retained as -is
COVID-19, Pneumonia and Normal Chest X-ray PA Dataset [22]	Pneumonia	Pneumonia	Retained as -is
	COVID-19	Other	Not part of target diseases
Chest X-Ray Images (Pneumonia) [23]	Pneumonia	Pneumonia	Retained as-is
Tuberculosis (TB) Chest X-ray Database [24], TBX11K [25], Belarus Dataset [26], Chest X-rays tuberculosis from India [27]	Tuberculosis	Tuberculosis	Retained as-is

* PadChest annotations comprise 174 NLP-derived radiographic findings; target disease labels in this study were assigned as positive if the matching condition was present in the PadChest findings. ** Shenzhen annotations are recorded in the findings column, including descriptors for conditions other than tuberculosis.

## Appendix C

To ensure the reliability of the uncertainty estimation reported in this study, we performed a justification and sensitivity analysis for the two key hyperparameters of the Monte Carlo Dropout (MCD) method: the dropout rate and the number of stochastic forward passes.

The dropout rate was set to 0.4. This selection strictly adheres to the original architectural design of EfficientNet-B5 proposed by Tan and Le [30] to balance model capacity and regularization. This value is also the established default in standard deep learning framework implementations of this architecture [37], ensuring that feature distributions remain consistent with the pre-trained weights used for transfer learning.

We determined the optimal number of forward stochastic passes (*N*) for inference by analyzing the convergence of the uncertainty metrics (Predictive Entropy and Variance). We evaluated the model predictions with 5, 10, 15, and 20 passes to identify the point where the estimation of epistemic uncertainty stabilized. As presented in Table A4, the Mean Predictive Variance converged significantly by 10 passes (3.75 × 10^−4^) and remained constant through 15 and 20 passes. Similarly, Predictive Entropy showed asymptotic stability between 15 and 20 passes. Based on this “diminishing returns” analysis, 20 stochastic passes were selected as the operating point. This ensures that the uncertainty trends reported in our results are attributable to data properties rather than stochastic estimation noise.

**Table A4:** Sensitivity analysis of uncertainty metrics with respect to the number of stochastic passes (*N*).

Stochastic Passes (*N*)	Mean Predictive Entropy	Mean Variance of Predictions	Relative Change (Variance)
5	1.408	3.13 × 10^−4^	-
10	1.142	3.75 × 10^−4^	+19.8%
15	1.146	3.75 × 10^−4^	0.0% (Converged)
20 (Selected)	1.146	3.75 × 10^−4^	0.0% (Stable)
